# Glutathione Metabolism Contributes to the Induction of Trained Immunity

**DOI:** 10.3390/cells10050971

**Published:** 2021-04-21

**Authors:** Anaisa V. Ferreira, Valerie A. C. M. Koeken, Vasiliki Matzaraki, Sarantos Kostidis, Juan Carlos Alarcon-Barrera, L. Charlotte J. de Bree, Simone J. C. F. M. Moorlag, Vera P. Mourits, Boris Novakovic, Martin A. Giera, Mihai G. Netea, Jorge Domínguez-Andrés

**Affiliations:** 1 Department of Internal Medicine and Radboud Center for Infectious Diseases (RCI), Radboud University Nijmegen Medical Center, 6500 HB Nijmegen, The Netherlands; Valerie.Koeken@radboudumc.nl (V.A.C.M.K.); Vasiliki.Matzaraki@radboudumc.nl (V.M.); Charlotte.deBree@radboudumc.nl (L.C.J.d.B.); Simone.Moorlag@radboudumc.nl (S.J.C.F.M.M.); Vera.Mourits@radboudumc.nl (V.P.M.); Mihai.Netea@radboudumc.nl (M.G.N.); 2 Instituto de Ciências Biomédicas Abel Salazar (ICBAS), Universidade do Porto, 4050-313 Porto, Portugal; 3 TWINCORE, a joint venture between the Helmholtz-Centre for Infection Research (HZI) and the Hannover Medical School (MHH), 30625 Hannover, Germany; 4 Centre for Individualised Infection Medicine (CiiM), Department of Computational Biology for Individualised Infection Medicine, a joint venture between the Helmholtz-Centre for Infection Research (HZI) and the Hannover Medical School (MHH), 30625 Hannover, Germany; 5 Center for Proteomics and Metabolomics, Leiden University Medical Center (LUMC), 2333 ZA Leiden, The Netherlands; s.kostidis@lumc.nl (S.K.); j.c.alarcon_barrera@lumc.nl (J.C.A.-B.); m.a.giera@lumc.nl (M.A.G.); 6 Epigenetics Research, Murdoch Children’s Research Institute, Parkville, VIC 3052, Australia; boris.novakovic@mcri.edu.au; 7 Department of Paediatrics, University of Melbourne, Melbourne, VIC 3052, Australia; 8 Department for Genomics & Immunoregulation, Life and Medical Sciences Institute (LIMES), University of Bonn, 53115 Bonn, Germany

**Keywords:** glutathione, trained immunity, macrophages, metabolism, innate immune memory

## Abstract

The innate immune system displays heterologous memory characteristics, which are characterized by stronger responses to a secondary challenge. This phenomenon termed trained immunity relies on epigenetic and metabolic rewiring of innate immune cells. As reactive oxygen species (ROS) production has been associated with the trained immunity phenotype, we hypothesized that the increased ROS levels and the main intracellular redox molecule glutathione play a role in the induction of trained immunity. Here we show that pharmacological inhibition of ROS in an in vitro model of trained immunity did not influence cell responsiveness; the modulation of glutathione levels reduced pro-inflammatory cytokine production in human monocytes. Single nucleotide polymorphisms (SNPs) in genes involved in glutathione metabolism were found to be associated with changes in pro-inflammatory cytokine production capacity upon trained immunity. Also, plasma glutathione concentrations were positively associated with ex vivo IL-1β production, a biomarker of trained immunity, produced by monocytes of BCG-vaccinated individuals. In conclusion, glutathione metabolism is involved in the induction of trained immunity, and future studies are warranted to explore its functional consequences in human diseases.

## 1. Introduction

Until recently, adaptive immunity was thought to be the only component of host defense characterized by the capacity to retain memory, tailoring T and B cell responses to a specific antigen. However, a growing body of recent research has also attributed characteristics of heterologous memory to the innate immune system, a process termed trained immunity [1]. Monocytes exposed for a short period of time to *Candida albicans*, the fungal cell wall component β-glucan, or even sterile stimuli as oxidized low-density lipoprotein (oxLDL), show enhanced production of pro-inflammatory cytokines upon an unrelated secondary stimulation long after the initial stimulus has been removed [2,3]. In addition, epidemiological studies have shown that the Bacillus Calmette-Guérin (BCG) vaccination against tuberculosis boosts the antimicrobial function of innate immune cells, subsequently inducing non-specific protection to unrelated infections and decreasing all-cause mortality [4].

Trained immunity is rooted in epigenetic changes that modulate the accessibility of genes of the pro-inflammatory response for the transcriptional machinery of the cell. Epigenetic rewiring in trained innate immune cells is accompanied by metabolic changes that promote pathways such as glycolysis, oxidative phosphorylation, and the cholesterol biosynthesis [5,6]. Metabolites derived from these pathways are signaling molecules and cofactors that can in turn modulate the activity of chromatin modifying enzymes. Glutathione (GSH) is the main antioxidant preserving the intracellular redox balance and has been suggested to contribute to epigenetic changes [7]. In addition, stimuli that induce trained immunity, such BCG and oxLDL, increase the production of reactive oxygen species (ROS) of macrophages upon secondary stimulation [8,9,10]. We hypothesized that the increased metabolic activity of trained macrophages would also increase ROS levels and modulate GSH, which in turn would influence the strength of trained immunity responses.

## 2. Materials and Methods

### 2.1. Isolation of Human Peripheral Blood Mononuclear Cells (PBMC) and Monocytes

Buffy coats from healthy donors were obtained after written informed consent (Sanquin Blood Bank, Nijmegen, The Netherlands). Ethical approval was obtained from the CMO Arnhem-Nijmegen (NL32 357.091.10). Isolation was performed by differential density centrifugation over Ficoll-Paque (GE Healthcare, Chalfont St Giles, UK). Subsequently, isolation of monocytes was performed with a hyper-osmotic Percoll (Sigma-Aldrich, St Louis, MO, USA) density gradient centrifugation and washed once with pyrogen-free cold phosphate buffered saline (PBS). Cells were resuspended and later cultured in RPMI 1640 Dutch modified medium (Invitrogen, Waltham, MA, USA) supplemented with 5 μg/mL gentamicin (Centraform, Etten-Leur, the Netherlands), 2 mM Glutamax (Gibco, Walthan, MA, USA), and 1 mM pyruvate (Gibco). To ensure maximal purity, Percoll-isolated monocytes were left to adhere to polystyrene flat bottom plates (Corning, Sigma-Aldrish, New York, NY, USA) for 1 h at 37 °C 5% CO_2_ and then washed once with warm PBS.

### 2.2. In Vitro Trained Immunity Model

Adherent monocytes were cultured as previously described [11]. Briefly, 10^5^ monocytes were seeded on flat-bottom 96-well plates (Greiner, Kremsmünster, Austria) and incubated with 1 µg/mL β-glucan or 5 µg/mL BCG vaccine (InterVax, Toronto, ON, Canada) in RPMI with 10% human pooled serum. β-1,3-(D)-glucan (β-glucan) from *Candida albicans* was kindly provided by Professor David Williams (College of Medicine, Johnson City, TN, USA). When indicated, cells were preincubated with 0.5µM diphenyleneiodonium (DPI), 50 µM a-tocopherol (AT) (Sigma-Aldrich), 0.5 µM ascorbic acid (AA), 1 mM N-acetyl cysteine (NAC) (Sigma-Aldrich), or 100 µM DL-buthionine sulfoximine (BSO) (Sigma-Aldrich) for 1 h prior to β-glucan addition. After 24 h, cells were washed once with warm PBS and left to differentiate in RPMI supplemented with 10% pooled human serum for 5 days. Medium was refreshed at day 3 of culture. At day 6, macrophages were restimulated with 10 ng/mL *Escherichia coli* lipopolysaccharide (LPS; serotype 055:B5, Sigma-Aldrich) for an additional 24 h.

### 2.3. Reactive Oxygen Species (ROS) Quantification

Monocytes/macrophages were cultured for 2 h, 24 h, or 6 days, washed, detached with cold PBS, and incubated with 5 µM H2DCFDA (Life Technologies, Waltham, MA, USA) in PBS for 30 min at 37 °C. Cells were analyzed by flow cytometry (CytoFlex, Beckman Coulter, Brea, CA, USA). Data analysis was performed using the Kaluza 2.1 software. Cell aggregates were gated out based on the forward scatter (FSC)-height versus FSC-area plot and the monocyte/macrophage population was gated according to FSC and side scatter (SSC). Data are presented as mean ± SEM and analyzed with Friedman’s test followed by Dunn’s multiple comparisons test. A *p* value below 0.05 was considered statistically significant, as indicated by asterisks (* *p* < 0.05).

### 2.4. RNA Sequencing Data of Human Monocytes Stimulated In Vitro

Gene expression analysis of monocytes exposed in vitro to β-glucan was performed using previously published RNA-seq data [12]. Briefly, PBMCs were isolated by centrifugation in Ficoll-Paque (GE Healthcare). Monocytes were purified using negative selection with beads for CD3+ (T cells), CD19+ (B cells), and CD56+ (NK cells) positive cells (Miltenyi Biotech, Bergisch Gladbach, Germany). Cells were cultured following the described in vitro trained immunity model and exposed to 5 μg/mL β-glucan. Monocytes were collected at 4 h and 24 h after exposure. Genes related to antioxidant response and glutathione metabolism were extracted from the list of significantly dynamic genes (*p* < 0.05, FC > 2.5, RPKM > 1) in the in vitro monocyte-to-macrophage trained immunity model. Data is presented as the log_10_ of the ratio between monocytes treated with β-glucan and non-stimulated monocytes.

### 2.5. Glutathione Quantification

Quantitative analysis of intracellular reduced (GSH) and oxidized (GSSG) glutathione was performed using nuclear magnetic resonance (NMR) spectroscopy as previously described [13]. Briefly, growth medium was removed and cultured cells were washed with warm PBS (37 °C) and quenched with liquid nitrogen to arrest metabolism. The cells were subsequently scraped off the plates and extracted using a cold (−80 °C) solution of methanol/chloroform/water, 8.1:0.9:1 (vol/vol/vol). The extracts were kept frozen on dry ice for at least 30 min and were subsequently centrifuged for 20 min at 18,000× *g* at −4 °C. The protein pellet was kept for protein quantification while the supernatants containing the intracellular metabolites were dried under a gentle stream of nitrogen and NMR samples were prepared by dissolving the dried material with 0.22 mL of 0.15 M phosphate buffer (pH 7.4) in deuterated water containing 0.05 mM trimethylsilyl propionic-d4-sodium salt as internal standard for NMR referencing and quantification. An 1D ^1^H NMR spectrum was collected for each sample on a 14.1 T (600 MHz for ^1^H) Bruker Avance II NMR, using the *noesygppr1d* pulse sequence (Topspin v3.0, Bruker Biospin Ltd, Karlsruhe, Germany). All spectra were processed and imported in Chenomx NMR suite 8.4 (Chenomx NMR suite, v8.0, Edmonton, AB, Canada) for the quantification of glutathione. All concentrations were normalized to the total protein mass of each sample. The latter was quantified by dissolving the protein pellet in buffer (1:1, 1% SDS: 8M Urea (0.25M Tris pH:8)) with sonication. The protein concentration was then measured using PierceTM BCA protein assay kit (Thermo Fisher Scientific, Waltham, MA, USA) according to its manual.

### 2.6. In Vitro and In Vivo Trained Immunity Models for Genetic and Metabolic Analysis

In the in vitro model, adherent monocytes were incubated either with culture medium only as a negative control, 2 μg/mL of β-1,3-(D)-glucan or 5 μg/mL BCG (300BCG cohort: BCG-Bulgaria strain, Intervax, Canada), for 24 h at 37 °C in the presence of 10% pooled human serum. On day 6, cells were re-stimulated for 24 h with either 200 μL RPMI or LPS 10 ng/mL (serotype 055: B5; Sigma-Aldrich). In the in vivo model, healthy adults were vaccinated with a standard dose of 0.1 mL BCG intradermally in the left upper arm, and blood was collected before and 14 and 90 days after vaccination. In each visit, 5 × 10^5^ PBMCs were stimulated with heat-killed *Staphylococcus aureus* (10^6^ CFU/mL) or left unstimulated at 37 °C with 5% CO_2_. Secreted cytokines were measured in both models after 24 h of stimulation, and the fold increase cytokine responsiveness induced by the training stimuli was used as a measure for trained immunity [14].

### 2.7. Genetic Analysis

Genetic analysis was performed using a cohort of healthy individuals of Western European descent from the Human Functional Genomics Project [15]. The 300BCG cohort consists out of 325 adults from the Netherlands (44% males and 56% females, age range 18–71 year). The study was approved by the local ethics committee CMO region Arnhem- Nijmegen, NL58553.091.16. DNA samples of individuals (*n* = 325) were genotyped using the commercially available SNP chip, Infinium Global Screening Array MD v1.0 from Illumina. Opticall 0.7.0 with default settings was used for genotype calling [16]. Samples with a call rate ≤ 0.99 were excluded, as were variants with a Hardy-Weinberg equilibrium (HWE) ≤ 0.0001, and minor allele frequency (MAF) ≤ 0.001. Strands of variants were aligned and identified against the 1000 Genome reference panel using Genotype Harmonizer [17]. We then imputed the samples on the Michigan imputation server using the human reference consortium (HRC r1.1 2016) as a reference panel, and we filtered out genetic variants with an R^2^ < 0.3 for imputation quality, and MAF < 5% [18], resulting in approximately 4 million single-nucleotide polymorphisms (SNPs) for follow-up QTL mapping. Both genotype and cytokine data on in vitro-trained immunity responses was obtained for a total of 267 individuals from the 300 BCG cohort. Three samples were excluded due to medication use (of which one was identified as a genetic outlier), and one sample due to onset of type 1 diabetes during the study. First, the fold change of cytokine production between trained and non-trained cells was taken as a measurement for the magnitude of the trained immunity response. Following quality check for cytokine distribution and after excluding genetic outliers, we mapped the log-transformed fold changes of cytokine production to genotype data using a linear regression model with age and sex as covariates to correct the distributions of fold change of cytokine production. We used a cutoff of *p* < 9.99 × 10^−3^ to identify suggestive quantitative trait loci (QTL) associations affecting trained immunity responses. Using the in vivo trained immunity model, both genotype and cytokine data was obtained for a total of 296 individuals. In addition to the outliers described for the in vitro QTL mapping, 18 evening vaccinated individuals were excluded, resulting in 278 samples before QTL mapping. First, raw cytokine levels were log-transformed and ratios of cytokine production between the visits were taken as the fold change of cytokine production. The fold change of cytokine production was mapped to genotype data using a linear regression model with age and sex as covariates. We used a cutoff of *p* < 9.99 × 10^−3^ to identify suggestive QTL associations affecting trained immunity responses. R-package Matrix-eQTL was used for cytokine QTL mapping [19].

### 2.8. Metabolomics Analysis

Metabolite levels of individuals from the 300 BCG cohort were measured before BCG vaccination. The metabolic features were measured and annotated by the General Metabolics (Zurich, Switzerland) using flow injection time-of-flight mass (flow-injection TOF-M) spectrometry [20]. Non-targeted metabolites were annotated according to human metabolites database (HMDB). All metabolomic measurements were performed in duplicates, and the average value was calculated for each sample per metabolite. Spearman correlation analysis was performed on metabolites and ex vivo cytokine fold changes in relation to before vaccination responses. To test the relation between glutathione levels and the SNPs of interest identified in the genetic analysis, a linear regression analysis with age and sex included as covariates was performed for each individual SNP.

### 2.9. Cytokine Quantification and Analysis

Cytokine production was determined using commercial ELISA kits for IL-1β, IL-6 and TNFα (R&D Systems, Minneapolis, MN, USA) following the instructions of the manufacturer. Data are presented as mean ± SEM and analyzed with two-way repeated measures ANOVA followed by Sidak’s multiple comparisons test. A *p* value below 0.05 was considered statistically significant, as indicated by asterisks (* *p* < 0.05).

## 3. Results

### 3.1. β-Glucan- and BCG-Induced Trained Immunity Trigger a Sustained Reactive Oxygen Species Production

**Figure 1:**
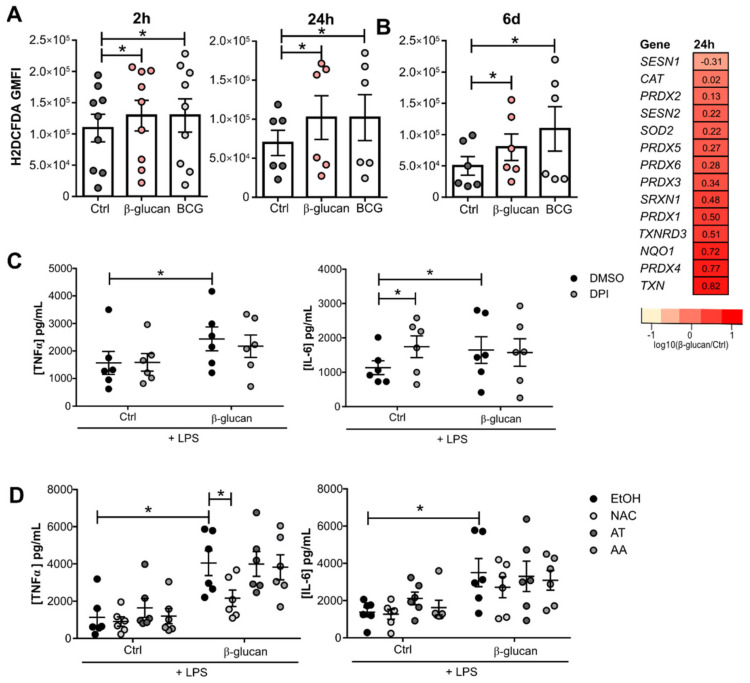
Increased ROS levels of trained monocytes do not contribute to their enhanced pro-inflammatory cytokine production. (**A**) ROS levels at 2 h, 24 h, and 6 days after exposure of monocytes to β-glucan and BCG (*n* = 6/9 donors, pooled from 2/3 independent experiments. Fridman test Dunn’s multiple comparisons test). (**B**) Expression levels of genes involved in the antioxidant defense in monocytes exposed to β-glucan for 24 h in comparison to unstimulated cells. Expression presented as log_10_(ratio). TNFα and IL-6 produced by β-glucan-trained macrophages after a 1 h pretreatment with (**C**) 0.5µM DPI (*n* = 6 donors, pooled from 2 independent experiments) and (**D**) 1 mM NAC, 50 µM AT or 0.5 µM AA (*n* = 6 donors, pooled from two independent experiments, two-way ANOVA, Sidak’s multiple comparisons test). (mean ± SEM, * *p* < 0.05).

Increased ROS release has been previously described as a feature of the trained immunity phenotype [21]. Monocytes exposed to β-glucan or BCG for 2 h and 24 h exhibit increased ROS. Interestingly, this increase was sustained and present after 5 days of resting in culture media (Figure 1A). Enhanced ROS levels are accompanied by transcriptional changes of different antioxidant genes in monocytes exposed to β-glucan for 24 h (Figure 1B). Mitochondrial superoxide dismutase *SOD2* and different thiol-containing molecules are upregulated, namely thioredoxin-dependent peroxide reductases 1-6 (*PRDX 1-6*) and sulfiredoxin 1 (*SRXN1*). The expression of thioredoxin (*TXN*) and thioredoxin reductase (*TXNRD*), relevant thiol-containing ROS scavengers, are also augmented.

However, neither the inhibition of the major ROS producer NADPH oxidase with diphenyleneiodonium (DPI), nor the addition of the antioxidant molecules α-tocopherol (AT) and ascorbic acid (AA) prior to incubation with β-glucan, modulated the increased macrophage responsiveness. In macrophages exposed to β-glucan, TNFα and IL-6 secretion 24 h after LPS restimulation were not modified by this pharmacological approach (Figure 1C,D). However, exposure to DPI alone increased IL-6 production upon LPS stimulation. (Figure 1C). Pretreatment with the general ROS scavenger N-acetyl cysteine (NAC) caused a decrease in TNFα and IL-6 production when compared to cells exposed to β-glucan alone (Figure 1D). Taken together, we show that increased ROS production plays a minor role in the increased cytokine production of β-glucan-induced trained immunity. In addition, trained immunity sustained increased ROS levels could be allied to the modulation of the cellular antioxidant glutathione, since NAC is not only a ROS scavenger but can also act as a precursor for glutathione synthesis.

### 3.2. Glutathione Metabolism Influences Trained Immunity Responses

**Figure 2:**
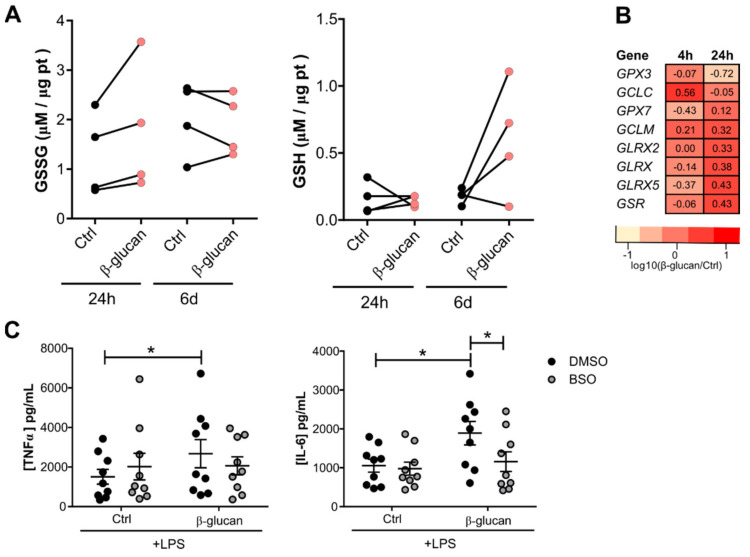
Glutathione levels are modulated upon β-glucan exposure. (**A**) Reduced and oxidized glutathione intracellular levels in monocytes after 24 h exposure with 1 µg/mL β-glucan and 5 days after the resting period (*n* = 4 donors, pooled from two independent experiments). (**B**) Expression of genes involved in glutathione metabolism in monocytes exposed to β-glucan for 4 h and 24 h. Expression presented as log_10_(ratio). (**C**) TNFα and IL-6 produced by β-glucan-trained macrophages after a 1 h pretreatment with 100 µM BSO (*n* = 9 donors, pooled from three independent experiments, * *p* < 0.05 two-way ANOVA, Sidak’s multiple comparisons test) (mean ± SEM).

Monocytes exposed to β-glucan for 24 h show higher levels of the oxidized form of glutathione (GSSG), which is in accordance with the higher ROS levels found at this time point (Figure 2A). After the resting period, the reduced form of glutathione (GSH) tends to be increased in trained macrophages, with no alteration of the concentrations of the oxidized form.

The genes that encode for the subunits of the rate-limiting enzyme glutamate cysteine ligase (*GCLC* and *GCLM*) show increased expression at 4 h after β-glucan stimulation compared to control monocytes. Glutathione reductase (*GSR*), glutathione peroxidase 7 (*GPX7*), and enzymes of the glutaredoxin family (*GLRX, 2, 5*) are also upregulated at the 24 h timepoint (Figure 2B), suggesting not only an increase in synthesis, but also a higher rate of glutathione recycling. Pharmacologically inhibiting the activity of GCL with buthionine sulfoximine (BSO) prior to β-glucan exposure leads to a decrease of IL-6 production after LPS restimulation (Figure 2C). Thus, exogenous modulation of glutathione levels, either supplementation by the addition of the precursor NAC, or its decrease by blocking the enzyme GCL with BSO, dampened the β-glucan increased responsiveness to secondary stimulation.

**Figure 3:**
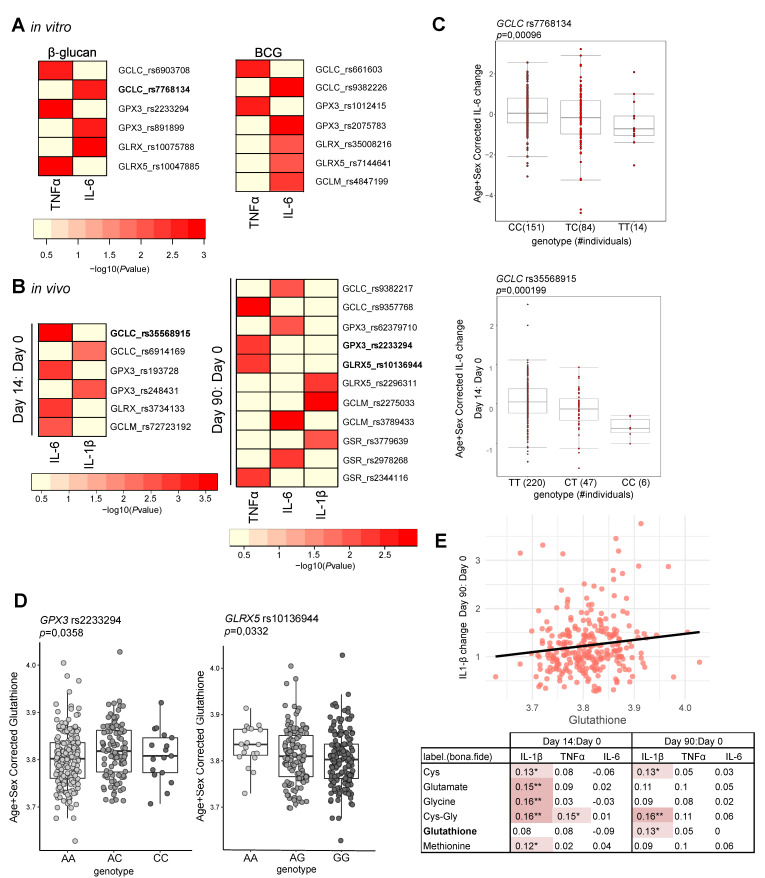
Glutathione is associated with trained immunity features. Heatmap of the *p*-values of association (*p* < 9.99 × 10^−3^) between SNPs mapped to genes involved in glutathione metabolism and the magnitude of cytokine production capacity by monocytes trained in vitro with (**A**) β-glucan and BCG (*n* = 251 healthy volunteers for IL-6 and *n* = 238 healthy volunteers for TNFα) and (**B**) in vivo BCG training responses (*n* = 278 healthy volunteers). (**C**) Boxplot of the lowest *p*-value SNP per analysis (rs7768134, rs35568915). (**D**) Boxplots showing glutathione plasma levels (corrected for age and sex) stratified by genotype for rs2233294 (*GPX3*) and rs10136944 (*GLRX5*, *n* = 302). The *p*-value for each SNP is derived from linear regression model with the SNP of interest as independent variable and glutathione (corrected for age and sex) as the dependent variable. (**E**) Spearman correlations between circulating metabolites at baseline involved in glutathione metabolism versus fold changes of PBMC-derived *S. aureus*-induced IL-6, IL-1β, and TNFα responses measured at 14 or 90 days after BCG vaccination compared to baseline (*n* = 297 healthy volunteers). The table presents the Spearman’s rho for each correlation, and the correlations in red highlight significant positive correlations (* *p* < 0.05, ** *p* < 0.01). The correlation between glutathione concentration and the fold change of IL-1β production at 90 days compared to day 0 is given as an example in a scatter plot.

To further investigate whether genetic variation in genes involved in glutathione metabolism influence trained immunity, QTL mapping was performed using a cohort of 325 healthy individuals. We tested if common SNPs with a minor allele frequency (MAF) > 0.05 in genes relevant for glutathione metabolism were associated to changes in pro-inflammatory cytokine production capacity of monocytes upon β-glucan and BCG in vitro training (Figure 3A,C). Similarly, we also assessed the impact of these genetic variants on the in vivo trained immunity induced by BCG vaccination of 278 healthy individuals (Figure 3B,C). Several SNPs within a window of 250 kb of genes involved in glutathione metabolism were suggestively associated (*p* value < 9.99 × 10^−3^) with up-regulation of IL-1β, TNFα, and IL-6 secretion after training (Figure 3A,B). In the same cohort, we measured the plasma metabolites involved in glutathione metabolism prior to BCG vaccination. SNPs identified in the in vitro and in vivo analyses were significantly associated to circulating glutathione concentrations (*p* value < 0.05) (Figure 3D). In addition, we found a positive association between plasma glutathione concentration and ex vivo IL-1β production 90 days after BCG vaccination upon in vitro exposure to the heterologous stimulus *Staphylococcus aureus* (Figure 3E). The up-regulation of IL-1β production by BCG vaccination is also positively associated with the circulating concentration of other metabolites involved in glutathione metabolism, such as methionine, cysteine, glutamate, and glycine (Figure 3E). Overall, genes involved in glutathione metabolism containing QTLs for innate heterologous responses, to LPS (Figure 3A) or to *S. aureus* (Figure 3B), and correlation between glutathione serum levels and ex vivo IL-1β production, both point to a role of this pathway in the establishment of trained immunity.

## 4. Discussion

Macrophages are plastic cells that adapt their phenotype to different environmental stimuli. Trained macrophages rely on a high energy metabolism, with increased glycolysis, TCA cycle, and oxidative phosphorylation, to mount a response with enhanced pro-inflammatory cytokine production [6,22]. In addition to cytokine production, oxidative burst with rapid ROS production is increased in restimulated BCG-trained macrophages [8]. Splenic neutrophils isolated from BCG-treated mice also show increased ROS production [23]. In line with the augmented metabolic rate and the increased capacity to mount an oxidative burst, we observed that monocytes and macrophages exposed to β-glucan or BCG exhibit an increase in basal ROS production. A similar rise in ROS release was previously reported in oxLDL-trained monocytes [9]. ROS have effector functions in the clearance of pathogens but can also mediate signal transduction [24]. Pharmacological inhibition of the ROS-producing enzyme NOX or treatment with antioxidant molecules α-tocopherol and ascorbic acid did not ablate the β-glucan increased cytokine production. However, the ROS scavenger NAC inhibited the production of TNFα enhanced by β-glucan, indicating a possible role of ROS in trained immunity.

Interestingly, NAC is not only an antioxidant molecule, but it also acts as a precursor of glutathione, the main endogenous antioxidant molecule. There are two major cellular redox systems, pyridine nucleotide, such as NAD+/NADH, and thiol systems, that include glutathione and thioredoxins. The NAD+/NADH ratio has been shown to be increased in β-glucan-exposed monocytes in a sustained manner after the differentiation period in trained macrophages [22], similarly to what we report for ROS levels. Here we show that GSH concentrations are also modulated in trained immunity. Most strikingly, the concentration of the GSH-reduced form is only enhanced in differentiated trained macrophages, while the levels of the oxidized GSSG are stable. This suggests that trained macrophages have increased GSH stores, which in contrast to NAD+/NADH do not partner with the sustained ROS production. The contribution of glutathione metabolism to trained immunity is further strengthened by genetic and metabolic analysis of a cohort of healthy individuals (300BCG). Common genetic polymorphisms within GSH-related genes were found to influence the magnitude of cytokine production upon in vitro and in vivo trained immunity (*p* < 9.99 × 10^−3^). In addition, two of these QTL loci, *GPX3* at *chr*5 and *GLRX5* at *chr*14, were found to be associated with the GSH plasma concentrations (*p* < 0.05).

GSH is an antioxidant molecule, but it is also known to regulate gene expression through different epigenetic mechanisms. GSH participates in post-translational modifications, as observed in the S-glutathionylation of the nucleosome protein histone H3, and is powered by the methionine pathway that ultimately leads to s-adenosyl methionine (SAM) production. In turn, SAM is the major methyl donor used for DNA and histone methylation [7]. The TCA cycle metabolite fumarate accumulates in trained macrophages, and its derivative monomethyl fumarate induces a trained immunity phenotype [5]. In astrocytes, acute exposure of the derivative dimethyl fumarate depletes intracellular GSH, but increases total GSH at later timepoints [25]. Trained immunity is anchored by epigenic modifications that confer long-term memory [26]. The herein observed increased GSH content of trained macrophages might facilitate the epigenetic rewiring necessary for the establishment of innate immune memory.

Here, we show that pharmacological modulation of glutathione and the redox status of the cell decrease macrophage’s heterologous response, as seen by the effect of BSO or NAC on β-glucan-enhanced cytokine production. In addition, in a cohort of healthy individuals, GSH metabolism is associated with trained immunity traits. The trained immunity mechanisms that are shaped by GSH metabolism remain to be further explored, but the potential involvement of both metabolic and epigenic landscapes makes this metabolic hub an exciting avenue to investigate in future studies. These insights contribute to the unraveling of the metabolic and epigenetic wiring of trained immunity, allowing for a better understanding of the innate immune impact on health and disease. Ultimately, a better understanding will help identify novel targets of therapy in diseases characterized by dysregulation of innate immune processes.

## Data Availability

RNA-sequencing data presented in this study are available in the NCBI Gene Expression Omnibus under the accession number GSE85243 at https://www.ncbi.nlm.nih.gov/geo/query/acc.cgi?acc=GSE85243 (accessed on 6 April 2021).
